# Humanizing the pediatric intensive care unit through new technologies and artificial intelligence

**DOI:** 10.3389/frhs.2026.1868374

**Published:** 2026-07-31

**Authors:** Jose Luis Unzueta-Roch, Andres Castillo-Sanz, Ana Moral-Larraz, Alberto García-Salido, Amelia Martínez de Azagra Garde

**Affiliations:** 1 Paediatric Intensive Care Unit, Hospital Infantil Universitario Niño Jesús, Madrid, Spain; 2 Paediatric Technological Innovation Department, Foundation for Biomedical Research of Hospital Niño Jesús, Madrid, Spain; 3 Paediatric Technological Innovation Department, Universidad Autónoma de Madrid, Madrid, Spain

**Keywords:** artificial intelligence, electronic health records, humanizing AI, pediatric intensive care, predictive medicine

## Abstract

**Objective:**

To conduct a narrative review about the impact that new technologies and artificial intelligence may have on healthcare delivery and humanization of care in pediatric intensive care units.

**Methods:**

A retrospective narrative review on electronic health records, artificial intelligence, and intensive care was performed. Subsequently, a narrative discussion was developed addressing the challenges and difficulties associated with the implementation of these technologies, focused on three main domains: electronic health records and clinical information systems, artificial intelligence solutions in intensive care units, and ethical conflicts and liability issues.

**Conclusions:**

Digital solutions exist that may help reduce professional burnout, such as transcription tools and automated electronic recording of physiological parameters. However, there are currently no validated predictive solutions based on artificial intelligence implemented in intensive care units. Collaboration among institutions is essential for the validation of clinical decision-support solutions.

## 1 Introduction

The technological innovation experienced by pediatric intensive care units (PICUs) in recent years has led to a substantial volume of real-time clinical information (1). The increasing number of technological devices and its capacity of generating high-fidelity databases (2) have made necessary efforts to integrate technical solutions. In this scenario, the artificial intelligence (AI) appears as an essential tool (3, 4) to evolve and support clinical decisions.

In this new era, paper-based records maybe insufficient or obsolete. It appears necessary their replacement with electronic systems that ensure proper data integration and real-time utilization (5). Automated acquisition of physiological variables also reduces the administrative burden on nursing staff caring for these patients (6). It is essential to advance the integration of these automated records into the electronic health record (EHR) as well as to incorporate AI-based automatic transcription tools that save time in the documentation of clinical notes (7).

Nowadays, numerous clinical decision-support tools have been published (3, 8, 9). They cover complex triage systems, parametric trend analysis, and advanced alerts that anticipate critical events. The literature and these AI-based systems are mainly derived from retrospective analyses and internal validation studies. Currently, no prospective tools evaluate triage systems or multiparametric clinical deterioration alerts. There are multiple initiatives under way, primarily focused on validating such tools. Intensive care societies are working on developing guidelines for the integration of artificial intelligence into critical care practice, with particular attention to ethical conflicts and the associated challenges. The future points toward delivering therapies centered on the individual rather than on predefined patient types or disease categories (9, 10). Moreover, real-time physiological data management enables real-time validation of treatments and therapeutic interventions.

In this narrative review, we analyze and discuss how automatized data acquisition, integration in electronic health records (EHR) and Artificial Intelligence (AI) and Machine Learning (ML) have the potential to transform Pediatric Intensive Care Units (PICUs), shifting the care model from reactive and data-centric to one that is proactive and ultimately patient-centered for the child and their family. We also address the essential ethical, privacy, and data security considerations.

## 2 Methods

This narrative review was based on a targeted review of the literature related to electronic health records, artificial intelligence technologies, and their application and impact within PICUs. A targeted literature review was conducted across PubMed, and Google Scholar, to ensure broad coverage and inclusion of high-quality sources.

The following keywords and Booelan operators were used “artificial intelligence in PICU,” or “electronic health records and physician burnout,” “vital sign documentation,” or “electronic database in PICU,” “predictive medicine,” and “artificial intelligence in healthcare.” We included peer-reviewed articles, published in English between 2012 and 2025, consensus statements, and guideline documents relevant to these definitions, and excluded non-English publications without available translations, single-patient case reports, and conference abstracts. Priority was given to sources with pediatric-specific data or broad clinical applicability. Additional relevant articles were identified through reference lists of key publications and recent consensus statements. Studies were selected based on their relevance to three predefined domains: 1) the implementation and utilization of electronic health records and clinical information systems in PICU; 2) artificial intelligence–based solutions applied in paediatric intensive care units; and 3) clinical frameworks addressing challenges, limitations, and potential solutions. The final group of included studies was organized into thematic categories encompassing electronic health records, predictive and personalized medicine approaches, and existing conflicts, barriers, and unresolved issues within the field. We would like to emphasize that this is a narrative and descriptive review, without a systematic approach.

## 3 Discussion

### 3.1 From data overload to human clarity

The PICU environment is characterized by a significant volume of real-time clinical information, generated by monitoring systems, mechanical ventilators, and smart pumps. The accurate integration and interpretation of this vast and dispersed data are crucial, but often overwhelm staff, potentially leading to the loss of relevant clinical information. AI emerges as a potential cognitive co-pilot, capable of processing this complex information in reduced time, freeing the human team from the exhausting task of constant surveillance.

#### 3.1.1 Three pillars of patient-centered care driven by AI

The integration of high-density databases and machine learning algorithms have the potential to transform and redefine pediatric care based on the next three pillars: freeing up human time for genuine care, prediction not reaction and personalized treatment.

1. **Freeing up human time for genuine care**

**Figure 1:**
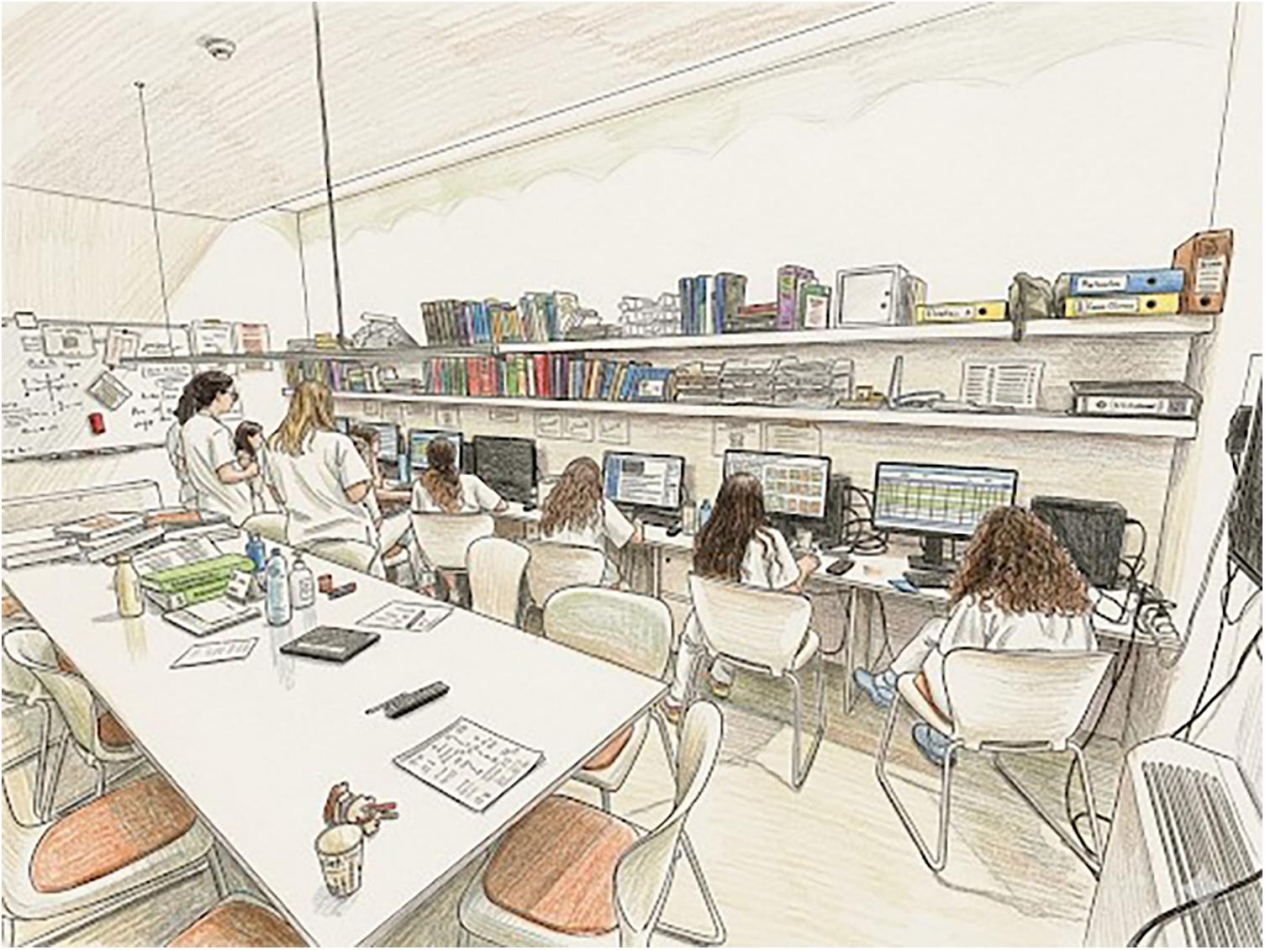
Artistic representation of the medical workspace. On certain occasions, the review of electronic health records must take place in a separate office, removed from the patient's immediate environment. An AI-assisted transformation (Gemini 3 Flash (April 2026 version)) was applied to the original authors' photograph to ensure the privacy and anonymity of the personnel and patients involved, without altering the structural context of the scene.

**Figure 2:**
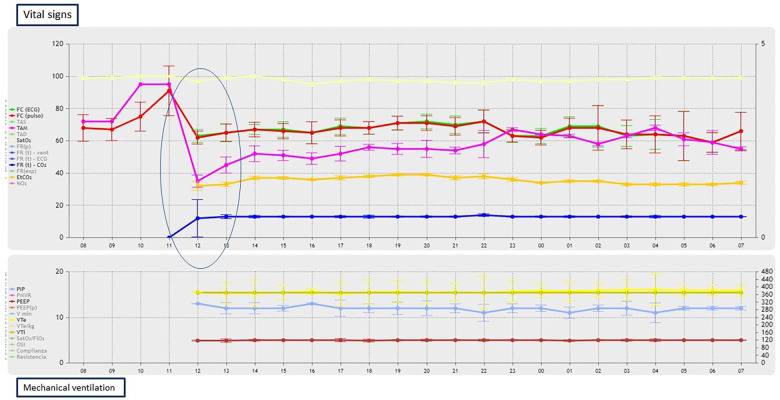
Real-time visualization of the impact of intubation and connection to mechanical ventilation on physiological vital signs in a real patient admitted in Pediatric Intensive Care Unit of Hospital Infantil Universitario Niño Jesús. Medlinecare® HDDCIS (medical online technology S.L, Valencia, Spain) high density data clinical information system.

The electronic health records documentation and its widespread adoption has resulted in health care safety, information accessibility but has become a major source of clinician and nurses burnout in many countries (7, 11). Time spent interacting with electronic devices is increasing through the years (12) (Figure 1). Patient data generated in the intensive care unit (ICU) has raised because of the new technical devices that can produce a huge amount of clinical data that normally was reduced to summary information. The most common data reduction strategies in clinical research rely in flowsheets and paper summary sheets recorded by bedside nurses and research coordinators, limitation in data storage and many missed data. This clear limitation has always been a risk discarding potential relevant information based on traditional descriptive statistics. Previous statistical model trying to predict physiological responses have been designed using those sparsed data, often each hour or even less frequently (13, 14), but acute critical patients and its clinical states and responses to treatment can variate in few minutes or even seconds. New bedside biomedical monitors and centralized computers are capable of acquiring vital signs each 5 s creating automatized high-frequency electronic databased in PICUs (5). These near continuous data storage models have relevant advantages as they identify brief events that could be missed in intermittent data collection methods. The time period window of data is crucial to estimate the patient's condition and to feed machine learning models when they are included in databases (13).Conventional electronic documentation has the potential to replace nurse-filled paper flowsheets, but these data needs to be human validated (15). Although monitoring technology and new devices have proliferated in critical care settings, only about a third integrate them with EHR and vital signs worksheet. Most of the medical devices are not designed to interoperate, but medical device standards to improve this interoperability is ongoing. Digital connections usually are though connection to serial (RS-232 or USB) or Ethernet (802.3) ports and some of them with wireless communications although this protocols normally are not compatible with electronic health records. Another critical point is time synchronization of the data for multiparametric and multimodal devices. It is impossible to interpretate the clinical information with the clinical devices if they are not in the same clock. These new advances have made possible to access data not previously available and reduce error attributable to clinical or nurse manual entry (16). The use of high-frequency data systems is the key to understand nor only intensive-care physiology but to individualize therapeutic action and the 4 main reason for that are: samplying frequency, multidevice capacity, information processing and ability to exploit information (10) (Figure 2).

Ambulatory nurses and physicians spent almost half of their days using EHR (6, 12). Even though there is no data about that in PICUs time spent in EHR is a major contribution to burnout (17, 18). The use of virtual scribes has demonstrated a decrease in total EHR time in medical specialists, but the greater time baseline EHR time the greater decrease in the proportion of time. There is no data analyzing this tool implementation in intensive care settings but it is a promising method that can achieve reduction in bureaucratic workload and centered patient attention (7).

**Figure 3:**
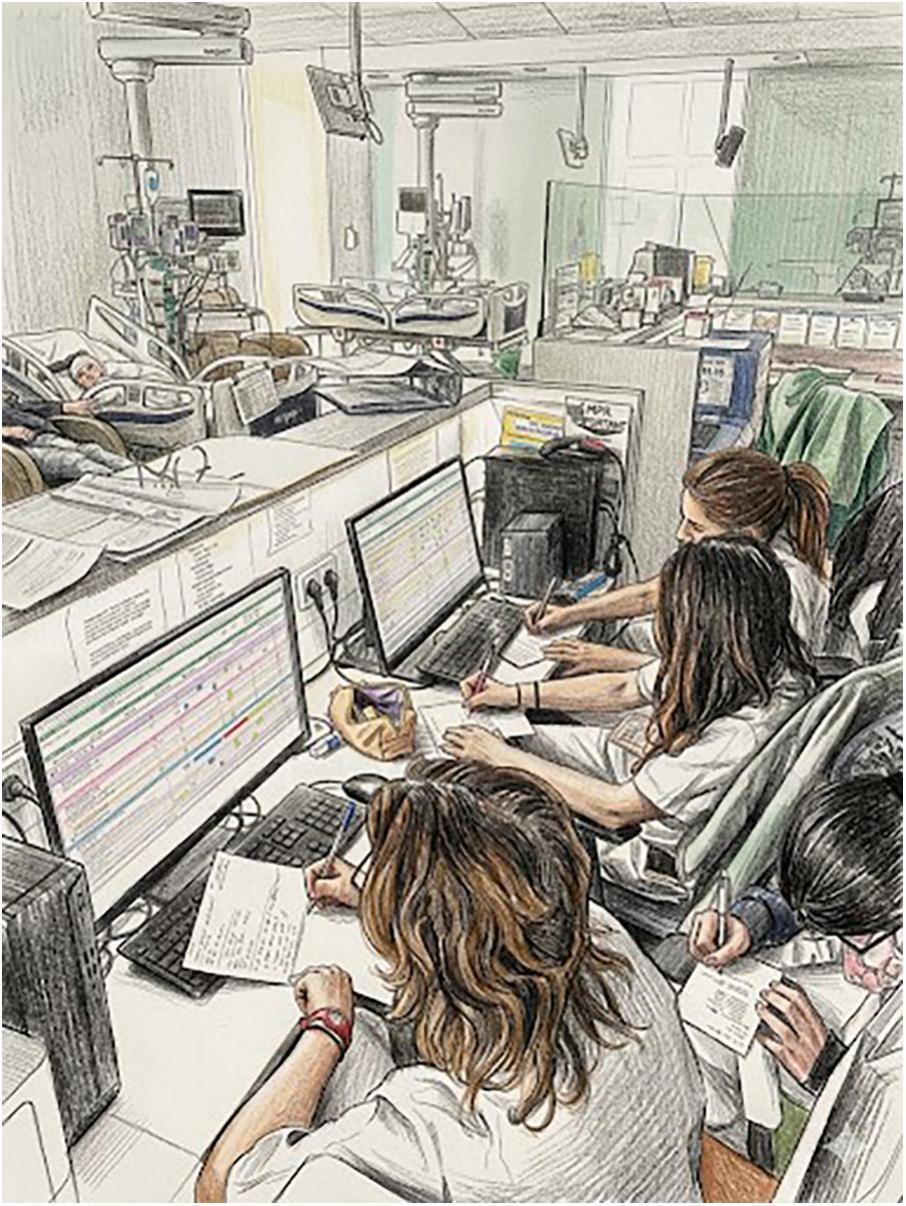
Artistic representation of the nurses workspace. The original photograph was captured by the authors and post-processed using AI-generative tools (Gemini 3 Flash) to convert it into a stylized illustration. This method was employed to ensure visual clarity while maintaining the original spatial composition.

New technological tools (multimodal automated clinical data entry and AI scribe systems) may enable nurses and medical staff to free up critical time, allowing them to devote greater attention to patient assessment and focus on patients' clinical status (Figure 3).

2. **Artificial Intelligence**

As early as 2022, Adegboro (3) stated that the introduction of artificial intelligence (AI) into healthcare represents a combination of hype and genuine hope. The impact of AI is grounded in the definition proposed by the European Institute of Innovation and Technology (EITH) in its report (19). The impact of AI on health outcomes was assessed by using a definition and focus area framework developed by the European Institute of Innovation and Technology (EIT) Health joint report. The primary objective should be to improve diagnostic performance or to enable physicians to dedicate more time to direct patient care and reduce burnout (20).

In another European report, Technology Readiness Levels (TRLs) are defined across nine categories. These categories represent the context in which a technological tool is being developed, its degree of maturity, and the environment in which it is ultimately implemented. Bedside AI applications may demonstrate their potential to improve health outcomes.

##### 3.1.1.1 Technology readiness levels (TRLs) in pediatric healthcare

Technology Readiness Levels (TRLs) provide a structured and widely accepted framework for evaluating the maturity, validation status, and clinical applicability of technological innovations. In the context of pediatric healthcare, and particularly in pediatric and neonatal intensive care units (PICUs and NICUs), TRLs are especially relevant due to the heightened safety requirements, ethical considerations, and vulnerability of the patient population.

Originally developed by the National Aeronautics and Space Administration (NASA) and later adopted by European research and innovation frameworks, the TRL scale ranges from TRL 1 (basic scientific observation) to TRL 9 (fully operational technology). This framework enables clinicians, researchers, and policymakers to objectively assess where a pediatric technology lies along the pathway from conceptual development to routine clinical use.

##### 3.1.1.2 Our conceptual proposal of the developmental phases applied to pediatrics

The nine TRL levels are commonly grouped into three phases—research, development, and deployment—which align well with the lifecycle of pediatric medical devices, digital health solutions, and artificial intelligence (AI) tools. We have proposed an adaptation of the Technology Readiness Levels (TRL) to the pediatric context (Table 1).

**Table 1:** Technology readiness level (TRL) adapted to pediatric frameworks.

Environment	TRL	Description	Pediatric framework
Research phase	TRL1	Basic principles observed	Fundamental biological, physiological, or computational principles relevant to pediatric populations are identified
	TRL2	Technology concept formulated	Potential pediatric applications are defined, such as AI algorithms tailored to age-adjusted vital signs, weight-based dosing, or developmental variability
	TRL3	Experimental proof of concept	Initial validation of the concept occurs, often through retrospective pediatric datasets, simulation environments, or small-scale exploratory studies
Development Phase	TRL4	Technology validated in laboratory settings	Components or prototypes are tested in controlled, non-clinical environments. For pediatric applications, this may include validation of algorithms using curated datasets that reflect pediatric age groups, disease prevalence, and physiological norms
	TRL5	Technology validated in relevant environments	The technology is evaluated in conditions that approximate real pediatric care settings, such as simulation labs, clinical decision-support testing, or pilot studies using real-world but supervised data.
	TRL6	Technology demonstrated in relevant pediatric environments	A functional prototype is tested within a pediatric clinical context, such as a PICU, NICU, or pediatric ward, under close monitoring
Deployment Phase	TRL7	System prototype demonstrated in operational pediatric settings	The technology is used in real clinical workflows, allowing assessment of performance, robustness, and acceptance by pediatric healthcare professionals
	TRL8	System completed and qualified	The technology reaches a high level of maturity, meeting regulatory, ethical, and quality standards specific to pediatric care
	TRL9	System proven in routine pediatric practice	The technology is fully implemented in everyday pediatric clinical care, with demonstrated benefits supported by clinical outcomes, real-world evidence, and longitudinal follow-up

###### 3.1.1.2.1 Research Phase (TRL 1–3): Foundational pediatric innovation

TRL 1—Basic principles observed: Fundamental biological, physiological, or computational principles relevant to pediatric populations are identified. At this stage, research may focus on age-specific physiology, developmental differences, or pediatric disease mechanisms without direct clinical application.

TRL 2—Technology concept formulated: Potential pediatric applications are defined, such as AI algorithms tailored to age-adjusted vital signs, weight-based dosing, or developmental variability. Early feasibility analyses and conceptual modeling are typically performed.

TRL 3—Experimental proof of concept: Initial validation of the concept occurs, often through retrospective pediatric datasets, simulation environments, or small-scale exploratory studies. In AI, this may involve training and testing models using anonymized pediatric electronic health records.

###### 3.1.1.2.2 Development phase (TRL 4–6): Validation in pediatric contexts

TRL 4—Technology validated in laboratory settings: Components or prototypes are tested in controlled, non-clinical environments. For pediatric applications, this may include validation of algorithms using curated datasets that reflect pediatric age groups, disease prevalence, and physiological norms.

TRL 5—Technology validated in relevant environments: The technology is evaluated in conditions that approximate real pediatric care settings, such as simulation labs, clinical decision-support testing, or pilot studies using real-world but supervised data.

TRL 6—Technology demonstrated in relevant pediatric environments: A functional prototype is tested within a pediatric clinical context, such as a PICU, NICU, or pediatric ward, under close monitoring. This stage is critical for identifying workflow integration challenges, safety issues, and clinician usability.

###### 3.1.1.2.3 Deployment phase (TRL 7–9): Clinical integration and routine use

TRL 7—System prototype demonstrated in operational pediatric settings: The technology is used in real clinical workflows, allowing assessment of performance, robustness, and acceptance by pediatric healthcare professionals.

TRL 8—System completed and qualified: The technology reaches a high level of maturity, meeting regulatory, ethical, and quality standards specific to pediatric care. This stage often includes compliance with medical device regulations and pediatric safety requirements.

TRL 9—System proven in routine pediatric practice: The technology is fully implemented in everyday pediatric clinical care, with demonstrated benefits supported by clinical outcomes, real-world evidence, and longitudinal follow-up.

###### 3.1.1.2.4 Importance of TRLs in pediatric and AI-based technologies

In pediatrics, the TRL framework is particularly valuable for preventing premature clinical adoption of immature technologies, especially artificial intelligence tools that may perform differently across age groups. Many pediatric AI solutions remain at intermediate TRLs due to challenges such as limited pediatric datasets, ethical concerns, and the need for age-specific validation.

By applying TRLs, stakeholders can systematically evaluate readiness, manage clinical risk, and align research investments with appropriate stages of technological development. Ultimately, the TRL framework supports the responsible translation of innovation into safe, effective, and equitable pediatric healthcare solutions (20).

##### 3.1.1.3 SPIRIT-AI/CONSORT-AI

The aim of the SPIRIT 2013 statement was to improve the completeness of clinical trial protocol reporting by providing evidence-based recommendations for the minimum set of items that should be addressed. More recently, it has been recognized that interventions involving artificial intelligence must undergo rigorous and prospective evaluation to demonstrate their impact on clinical outcomes.

The SPIRIT-AI extension is a new guideline for reporting protocols of clinical trials that evaluate interventions incorporating an AI component (21, 22). This guideline was developed in parallel with its complementary statement for clinical trial reporting, CONSORT-AI. Both extend existing clinical trial standards by introducing AI-specific requirements, including descriptions of the instructions and competencies required for their use, the setting in which the AI intervention will be implemented, considerations for handling input and output data, the interaction between humans and AI, and the analysis of error cases.

The key contribution of these guidelines is that they ensure transparency, reproducibility, and methodological rigor in clinical trials.

The following table summarizes the structural differences according to the framework's nature, objective, scale, focus, and output (Table 2).

**Table 2:** Structural differences between TRL and CONSORT-AI/SPIRIT-AI.

Dimension	TRL (AI healthcare)	CONSORT-AI / SPIRIT-AI
Nature	Maturity framework	Reporting guidelines
Objective	Assess readiness and deployment stage	Standardize trial design/reporting
Scale	9 levels (TRL 1–9)	Checklists (15+ AI-specific items)
Focus	Technology lifecycle	Clinical evidence quality
Output	Stage classification	Transparent publications

An appropriate way to compare these frameworks would be to align them. In this approach, TRL levels 1–3 would not require the application of CONSORT-AI/SPIRIT-AI guidelines. At intermediate levels (TRL 4–6), SPIRIT-AI becomes relevant, particularly for protocol design, definition of data pipelines, and specification of the target population. In contrast, at advanced levels (TRL 7–9), which include real-world validation, CONSORT-AI becomes essential for reporting the results of randomized clinical trials, evaluating efficacy, and characterizing bias and generalizability.

Based on this, it can be concluded that both frameworks are compatible; however, neither original TRL framework nor SPIRIT-AI/CONSORT-AI is specifically tailored to pediatric populations.

Artificial intelligence (AI) has the potential to transform clinical decision-making and optimize bedside care, thereby demonstrating improvements in health outcomes (23). Despite the fact that several years have passed since the systematic review conducted by Adegboro and that many stakeholders believe AI will change the future of healthcare delivery, rigorous safety controls are still required before these AI-based solutions can become part of physicians' routine clinical practice (8).

Unfortunately, the implementation of these solutions in the pediatric setting lags behind adult care, mainly because stricter safety requirements and stronger evidence of efficacy are demanded in this population. In fact, it has recently been documented that pediatricdevices require longer periods to receive approval from regulatory institutions.

Although many years have elapsed since the emergence of the first AI tools, solutions applicable to low-income countries have not yet been adequately developed, particularly in settings where intensive care unit bed management and financial resources are more limited.

The use of Technology Readiness Levels (TRLs) allows discrimination between artificial intelligence solutions that are more likely to be successfully implemented in intensive care units. It is important to highlight that many of the algorithms and AI-based solutions developed to date rely primarily on retrospective data. Therefore, there is a clear need to initiate prospective and experimental studies capable of measuring the real clinical impact of AI interventions.

The heterogeneity of the metrics currently used to assess the impact of AI limits the ability to compare different models and hampers the generation of robust, generalizable evidence. Furthermore, when translating algorithms designed and trained in a specific geographical region, it is essential to consider that they have been developed for a particular population. In many cases, these technological solutions lack adequate geographic or population-based validation, which may significantly affect their performance and safety when applied in different clinical and demographic contexts.

##### 3.1.1.4 Barriers

Artificial intelligence technologies must be capable of deciphering and translating millions of gigabytes of clinical data in order to function as clinical decision support systems and assist clinicians in real time. However, most of this information is stored in an unstructured format, making the interpretation of clinical notes highly complex, particularly during model training. As a result, the development and deployment of AI systems may require excessive energy and computational resources. In addition, information technology infrastructures are often highly heterogeneous across institutions, further increasing complexity.

The lack of data standardization and the coexistence of multiple health information systems substantially increase the energy consumption associated with Natural Language Processing (NLP), which is both costly and time-intensive. Moreover, when AI systems rely heavily on free-text clinical notes, the extracted information may be inaccurate or incomplete, potentially leading to erroneous outputs and negatively affecting patient safety.

Looking ahead, it is essential to measure healthcare professionals' level of trust in AI systems and to evaluate how their adoption impacts clinicians' daily practice, workload, and overall professional experience.

To ensure the safe development and high quality of AI models, researchers should prioritize the integration of specific guidelines, such as the pediatric-specific ACCEPT-AI framework (4, 24). ACCEPT-AI is a framework based on ethical principles that provides key recommendations for the use of pediatric data and the assessment of age-related algorithmic bias in AI/ML research for researchers, regulators, policymakers, and clinicians. It is designed for independent ethical use and can be integrated with other guidelines such as CONSORT-AI and SPIRIT-AI.

Hospitals should invest in local systems that function as a general landing zone where new models can be integrated quickly. Such a centralized system could ease the transition from isolated models to clinically usable applications. In addition, it would facilitate the monitoring and maintenance of all AI models.

A new step-by-step approach has been proposed to improve the quality, safety, and transparency of AI research (25).

3. **Personalized and predictive vs. reactive treatments**

Traditional healthcare models in the PICU predominantly operate on a reactive basis, intervening after symptoms manifestation. In contrast, predictive medicine seeks to anticipate disease susceptibility and progression through patient-specific data, exemplifying a paradigm shift towards proactive health management. The P4 Medicine framework underpins predictive healthcare, emphasizing Predictive, Preventive, Personalized, and Participatory strategies. Integrating predictive with reactive care ensures both immediate disease management and long-term risk reduction, optimizing individual and population health outcomes (1, 10).

Reactive treatments include pharmacologic therapy, surgical procedures, and physical rehabilitation, which primarily aim to manage extant disease rather than prevent it (26). While essential for acute care, reactive strategies are limited in preventing chronic disease progression and often incur higher long-term costs due to readmissions and complications.

Advantages of predictive treatments are early detection, personalized care, improved outcomes, cost efficiency, patient empowerment (26), but there are still some challenges about data requirements, risk of overdiagnosis, ethical concerns regarding genetic privacy, and patient compliance issues (27).

Reactive treatments have the known advantages, such as immediate symptom control, clear clinical protocols, critical for emergency interventions, among limitations on the inability to prevent disease onset, increased long-term costs, reduced opportunity for early intervention (10).

By exploiting high-dimensional datasets and integrating heterogeneous data streams from electronic health records (EHRs), medical imaging, genomics, and wearables and other IoT devices for physiological monitoring, AI ML and DL algorithms can detect latent patterns that are often indiscernible to human clinicians (1), identify complex correlations, and generate predictive models for disease onset and progression (28). Convolutional neural networks (CNNs) and recurrent neural networks (RNNs) have demonstrated efficacy in imaging-based diagnostics and temporal health assessments, respectively. These predictive capabilities enable the tailoring of individualized interventions also in the PICU, although there is currently no evidence of its use in practice.

Advances in the application of AI are improving the field of medical diagnosis, where a small revolution is taking place. It is being introduced as a diagnostic tool that appears to enhance accuracy, speed, and effectiveness in the diagnostic process of diseases (29). To achieve this, AI algorithms can interrelate data from medical records, complementary tests, and treatments. Improvements in the diagnosis of pediatric asthma have been demonstrated through the use of clinical data from medical records, as well as in the management of pediatric obesity (30, 31).

In the field of pediatric cardiology, algorithms have been developed for the early detection of potential complications by integrating vital signs and clinical data from medical records (32). It would be interesting to investigate the cross-applicability of these algorithms in pediatric intensive care units.

If multiparametric integration of monitoring systems is achieved—combined with a high density of clinical data and interoperable electronic health records—clinical decision-support solutions could be developed. Furthermore, if AI is implemented as an automated system for managing administrative tasks, it may enhance healthcare professionals' efficiency and help refocus priorities on direct patient care.

4. **Ethical Issues, challenges, bias and liability**

In many recent studies and throughout the literature, the PROBAST tool has been used to detect bias. However, this tool was originally developed for predictive models and not specifically for AI. Recently, a dedicated tool, PROBAST-AI, has been under development to address this limitation (33, 34).

In 2025, Cecconi et al. (35) published a consensus statement on the implementation of AI in intensive care. Although this consensus focuses on adult critical care, it highlights many of the reflections and concepts discussed above. It represents a call for community action to reduce the existing gap between technological innovation and patient-centered care. The group's objective was to facilitate a smooth transition toward personalized medicine while avoiding bias, maintaining equity in resource allocation and decision-making, promoting information sharing, and strengthening education to ensure the responsible use of AI.

One of the major ethical debates surrounding AI in healthcare concerns the assumption of an inherent benefit of algorithmic recommendations and the potential weakening of the human connection at the centre of healthcare delivery—a tradition that is millennia old and dates back to the origins of medical practice.

Algorithm development must therefore be undertaken from a human-centered perspective, promoting an empathetic approach that enables clinicians to spend more time at the patient's bedside engaging in communication and interaction. Moreover, some algorithms are developed outside the medical domain and are not bound by the principles of the Hippocratic Oath. For this reason, the creation of multidisciplinary teams—including physicians, patients, and technology experts—is strongly advocated. These teams would be responsible for supervising algorithm behavior, identifying potential risks, and promoting transparency in decision-making.

Another highly relevant consideration is that uncertainty is an intrinsic element of medical practice, to which clinicians have learned to adapt. At present, AI systems are limited in their ability to manage such uncertainty; however, the emergence of explainable AI aims to help bridge this gap.

Standardized data collection is essential for the development of generalizable and reproducible AI models. In intensive care units, data variability is particularly pronounced due to the wide range of sources involved, including bedside monitors, laboratory results, and medication records. Consequently, data integration is a critical requirement for the development of effective AI-based clinical decision support systems. Hospitals, technology developers, and institutions should therefore promote the adoption of communication standards that enable interoperability across systems.

As children and young patients under 18 years of age are underrepresented in scientific studies, algorithmic bias may arise from the generalization of results based on adult populations. This is known as “age-related algorithmic bias.” Pediatric health encompasses multiple developmental stages from birth through adolescence, and the incidence, prevalence, symptoms, and prognosis of diseases all vary according to age. The generation of pediatric data poses significant ethical and practical challenges, as patient recruitment differs substantially from that of adults, requiring parental or guardian consent, and strict data protection measures. The U.S. Department of Health and Human Services mandates special protections for children and young people (CYP) involved in research, establishing standards that are more stringent than those applied to adults (24). Collaboration among AI models should also be encouraged. Centralized data aggregation from multiple pediatric critical care units, as well as federated learning approaches within structured networks, enhance external validation and model reliability—both of which are essential for testing AI tools and cannot be achieved within a single center (36, 37). Federated learning is gaining increasing relevance in Europe, where data protection regulations adopt a more risk-averse stance toward AI development and favour decentralized solutions. In parallel, synthetic data generation is emerging as a complementary strategy that enables data sharing while preserving patient privacy.

Despite ongoing efforts to regulate AI in medicine, regulation itself remains one of the main barriers to its widespread implementation. By involving legal authorities, healthcare professionals, institutions, and patients, discussions about the role of AI in intensive care can move beyond governmental settings and closer to the patient's bedside, ultimately enhancing patient safety (38, 39).

In the United States, the main regulatory framework governing AI applications is their classification as medical devices under the FDA. The legal approach is product-based rather than technology-based. There are also Good Practice guidelines, although these are not always legally binding. Additionally, there is no specific regulatory framework for pediatric AI. Protection in this area largely depends on ethical initiatives and clinical studies.

In Asia, there is no single unified model; instead, the regulatory landscape is fragmented. In China, regulation is sector-specific, mandatory, and highly stringent, including strict data protection laws such as the Personal Information Protection Law (PIPL). In contrast, Japan has a hybrid regulatory system that combines medical device law for Software as a Medical Device (SaMD), data protection legislation (APPI), and the AI Promotion Act, which is non-binding. In South Korea, there is a framework AI law that imposes obligations on high-impact AI systems, aiming to balance innovation and safety.

The European Union (EU) took a major step toward regulating artificial intelligence (AI) with the adoption of the AI Act by its 27 Member States on March 13, 2024 (40).As the world's first comprehensive legal framework specifically dedicated to AI, the AI Act seeks to promote human-centered and trustworthy AI while safeguarding the health, safety, and fundamental rights of individuals against the potential risks posed by AI-enabled systems.

In Europe, AI systems that are used as, or integrated into, medical devices must comply simultaneously with the AI Act and the Medical Device Regulation (MDR). This dual regulatory requirement imposes stringent obligations on developers, including the need to demonstrate robust clinical evidence and ensure safety and performance across targeted populations. This is particularly relevant in pediatrics, where patients present distinct physiological and developmental characteristics compared to adults, requiring dedicated validation and evidence tailored to pediatric populations.

The AI Act is based on a risk-based classification system, which prioritizes transparency and prohibits unacceptable uses of artificial intelligence. This framework establishes a strict regulatory structure designed to ensure that AI applications are safe, transparent, and respectful of fundamental human rights.

Artificial intelligence applications in healthcare are generally classified as high-risk systems, which entails strict regulatory obligations. These include requirements for high-quality datasets, traceability in documentation, and conformity assessment procedures prior to deployment. Although the AI Act does not include a dedicated pediatric section, it explicitly mandates the protection of vulnerable groups, among which children are specifically included. In addition, data protection legislation imposes very strict rules regarding the processing of minors' personal data.

Pediatric patient data must therefore be handled with particular care, ensuring compliance with stringent standards for training data to prevent bias or discrimination against younger or vulnerable populations. Under the General Data Protection Regulation (GDPR), children are entitled to enhanced protection with respect to their personal data, as they may be less aware of the risks and implications of data processing. Organizations that handle pediatric health data or provide digital services to minors must use clear, age-appropriate language, obtain parental consent when required below the national age threshold, and always prioritize the best interests of the child.

###### 3.1.1.4.1 Core pediatric GDPR principles

- Age of Digital Consent: For online services (such as telehealth platforms or patient portals), parental consent is required if the child is below the nationally defined age threshold. Within the EU, this threshold ranges from 13 to 16 years, depending on the Member State (for example, 14 years in Spain and 16 years in Ireland).
- Special Category Data: Health and medical data are classified as special category data. Their processing requires a valid legal basis under Article 9 of the GDPR, such as the provision of medical care, preventive medicine, or public health purposes.
- “Best Interests of the Child” Principle: Any processing of a minor's personal data must be designed and carried out with the child's best interests as a primary consideration.
- Right to be Informed: Children have the same fundamental rights as adults to access, rectify, and erase their personal data. Privacy notices and consent forms must be presented in clear, simple, and age-appropriate language.
- Clinical Trials and Research: Pediatric clinical research must support minors in making informed decisions. This requires tailored documentation explaining clearly how their data will be collected, stored, and shared, taking into account their level of understanding.

Regulation (EU) 2025/327 on the European Health Data Area enables stronger, safer, and more ethical pediatric research by providing standardized access to electronic health data across the EU. It balances public health benefits, research innovation, and patient privacy, creating an environment that advances pediatric science, improves health outcomes, and promotes cross-border collaboration.

## 4 Conclusions

Although AI represents a highly promising technology, most current solutions remain confined to the research setting and have not yet been fully integrated into routine clinical practice. Despite being fueled by ICU data, insufficient time and infrastructure exist to support the effective bedside implementation of AI tools, and many authors tend to overestimate their technology readiness level (TRL). In pediatric care, physiological variability, data privacy concerns, and technical challenges further complicate widespread adoption. It is therefore our responsibility to pursue solutions that help re-humanize healthcare by reducing time spent on administrative tasks imposed by electronic health record systems—a major contributor to clinician burnout—and by developing alert and diagnostic systems that anticipate critical situations while enabling personalized treatments and care.

### 4.1 Limitations

This article does not constitute a systematic review, nor does it aim to do so. This decision reflects a conscious acknowledgment of the intrinsic difficulty of continuously updating the scientific evidence in a field that evolves at an extraordinary pace. The rapid generation of new data, models, and technological solutions—particularly in artificial intelligence—makes it methodologically challenging, if not unrealistic, to maintain a near–real-time synthesis of the available literature.

Moreover, there is an inherent temporal mismatch between technological innovation and clinical validation. From the initial conceptualization of a healthcare technology to its rigorous evaluation, clinical validation, and eventual regulatory approval, several years may elapse. In contrast, the technological ecosystem evolves at a much faster rate, with new tools, algorithms, and platforms emerging almost weekly. As a result, by the time a technology has been adequately assessed and its clinical benefits or limitations are clearly demonstrated, it may already be outdated or replaced by more advanced solutions.

This dynamic raises important questions regarding the traditional paradigms of evidence generation and evaluation in medicine. While rigorous validation remains essential to ensure patient safety and clinical effectiveness, overly prolonged evaluation cycles may inadvertently hinder the timely adoption of potentially beneficial innovations. Consequently, there is a need to strike a careful balance between methodological rigor and adaptive evaluation strategies that are better aligned with the pace of technological change, particularly in fields such as clinical and pediatric artificial intelligence.

## Data Availability

The original contributions presented in the study are included in the article/supplementary material, further inquiries can be directed to the corresponding author/s.
